# Gerbode defect resulting from Group B *Streptococcus* infective endocarditis: a case report

**DOI:** 10.1186/s40792-024-01943-5

**Published:** 2024-06-19

**Authors:** Kazuki Hisatomi, Tatsuya Miyanaga, Takashi Miura, Kiyoyuki Eishi

**Affiliations:** 1 Department of Cardiovascular Surgery, Nagasaki University Hospital, 1-7-1 Sakamoto, Nagasaki City, Nagasaki 852-8501 Japan; 2 Department of Cardiovascular Surgery, Hakujyuji Hospital, Fukuoka, Japan

**Keywords:** Infective endocarditis, Gerbode defect, Group B *Streptococcus*

## Abstract

**Background:**

Gerbode defect is an unusual abnormal communication between the left ventricle and the right atrium and is a serious complication of aortic infective endocarditis. Group B *Streptococcus* is an uncommon cause of infective endocarditis and has a markedly destructive effect on valvular tissue. Acute fistulation between the left ventricle and the right atrium associated with this form of infective endocarditis is a life-threatening, aggressive complication that often requires urgent surgical intervention. However, the identification of actual communication is often extremely difficult. Herein, we describe an unusual case of Gerbode defect resulting from Group B *Streptococcus* infective endocarditis and discuss the issues surrounding such a rare cardiac defect and such an infection.

**Case presentation:**

A 60-year-old man with underlying uncontrolled diabetes mellitus underwent endoscopic retrograde biliary drainage for acute cholangitis. On the 10th postoperative day, the patient developed multiple acute cerebral embolisms. Transthoracic echocardiography demonstrated severe aortic regurgitation and a large mobile vegetation near the tricuspid annulus. No obvious fistula between the left ventricle and the right atrium could be demonstrated. The blood culture examination was positive for Group B *Streptococcus*. The patient was diagnosed with Group B *Streptococcus* infective endocarditis, and antibiotic therapy was initiated. Transesophageal echocardiogram performed after referral to our hospital confirmed detachment of the right coronary cusp of the aortic valve from the annulus and an abnormal cavity immediately below the right coronary cusp. Color Doppler imaging finally revealed systolic blood flows from the left ventricle into the right atrium through the cavity. Therefore, we diagnosed the patient with Gerbode defect resulting from Group B *Streptococcus* infective endocarditis. In addition to aortic valve replacement, defect closure and left ventricular outflow tract repair were successfully performed urgently for severely complicated and uncommon infective endocarditis. The patient was uneventfully discharged without any complications.

**Conclusions:**

We reported successful surgical treatment of unusual active IE and Gerbode defect caused by GBS. Careful preoperative echocardiographic work-up is imperative for accurate early diagnosis and successful repair.

## Background

Gerbode defect is a rare abnormal communication between the left ventricle (LV) and the right atrium (RA), most often seen as a congenital defect or in association with destructive aortic valve infective endocarditis (IE). Although Group B *Streptococcus* (GBS) is an uncommon cause of IE, it has been increasingly reported as a cause of IE with an aggressive course and high mortality rate [1]. Early surgical management is the main treatment modality to correct the potentially serious complications of this form of IE. However, the identification of actual communication is often extremely difficult. Herein, we describe an unusual case of a 60-year-old man with Gerbode defect resulting from GBS active IE.

## Case report

A 60-year-old man was initially admitted to the hospital with acute cholangitis due to common bile duct stones. Endoscopic retrograde biliary drainage was performed successfully, but ataxic gait was observed on the 10th postoperative day. Brain magnetic resonance imaging showed multiple acute cerebral embolisms. Transthoracic echocardiography (TTE) demonstrated severe aortic regurgitation (AR) and a large mobile abnormal structure near the tricuspid annulus. No obvious fistula between the LV and the RA could be demonstrated. The patient was suspected of having IE and transferred to our department for treatment. The patient was hemodynamically stable (blood pressure: 106/43 mmHg, heart rate: 80 beats/min), and an electrocardiogram revealed normal sinus rhythm. However, the patient was febrile (38.0 °C) with respiratory distress. A chest X-ray showed cardiomegaly, pulmonary congestion, and massive pleural effusion. The blood culture examination was positive for *Streptococcus agalactiae* and antibiotic therapy was initiated. Relevant medical history included type 2 diabetes mellitus with poor blood glucose control (HbA1c: 11.2%).

**Fig. 1:**
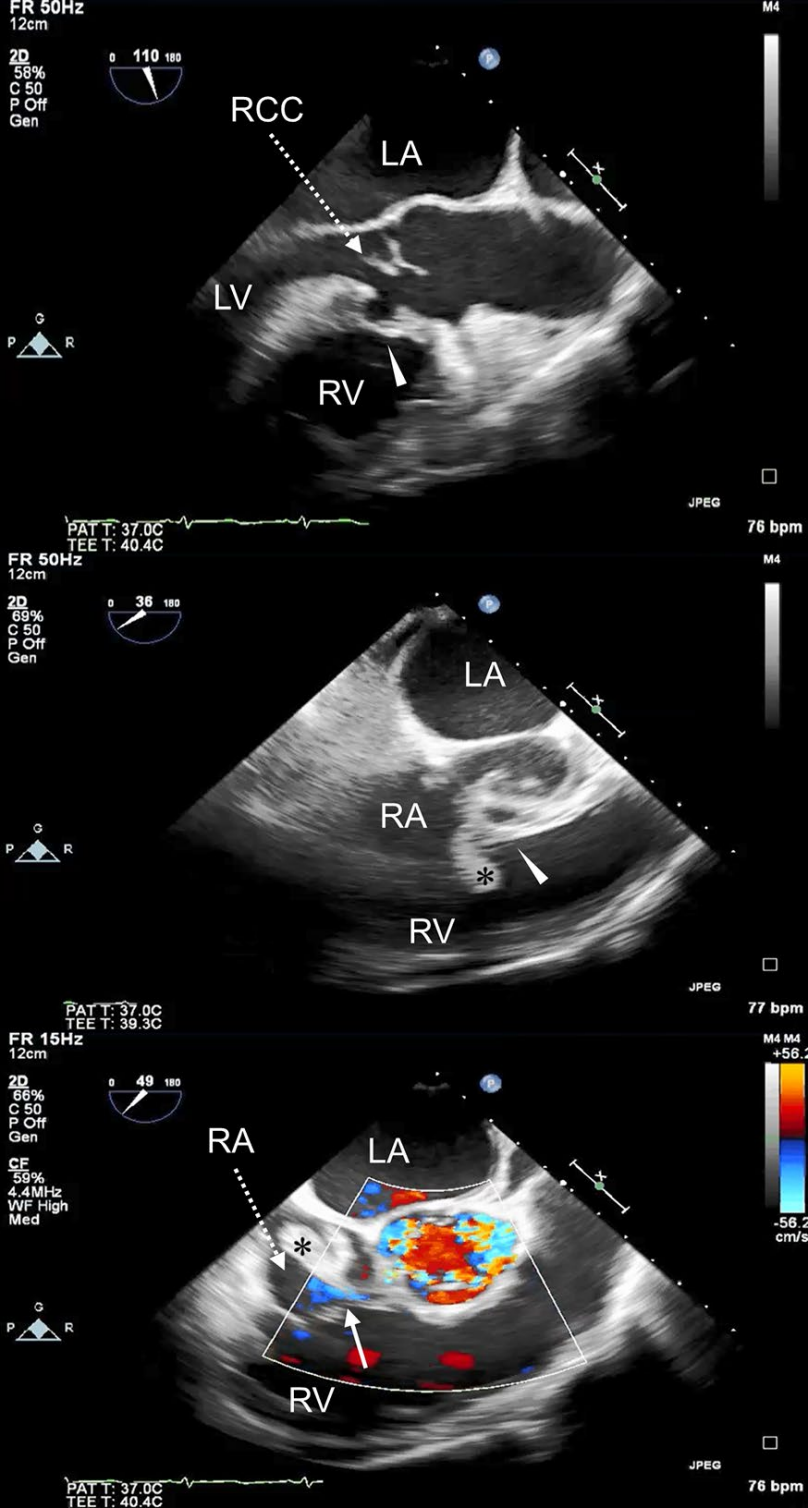
Preoperative TEE demonstrating detachment of the right coronary cusp of the aortic valve from the annulus and the cavity immediately below the right coronary cusp (white arrowhead). The vegetation was detected on the atrioventricular septum (black asterisk). Color Doppler imaging demonstrating systolic blood flows from the LV into the RA through the cavity (white arrow). LA: left atrium; LV: left ventricle; RA: right atrium; RV: right ventricle; RCC: right coronary cusp

Transesophageal echocardiogram (TEE) confirmed detachment of the right coronary cusp of the aortic valve from the annulus with severe AR. An abnormal small cavity immediately below the right coronary cusp was also detected. A hyperechoic mobile structure was found attached to the atrioventricular septum without tricuspid regurgitation. Further careful TEE was performed, and color Doppler imaging finally revealed systolic blood flow from the LV into the RA through the cavity (Fig. 1). Therefore, we diagnosed the patient with *S. agalactiae* active aortic IE and LV–RA communication known as an acquired Gerbode defect.

**Fig. 2:**
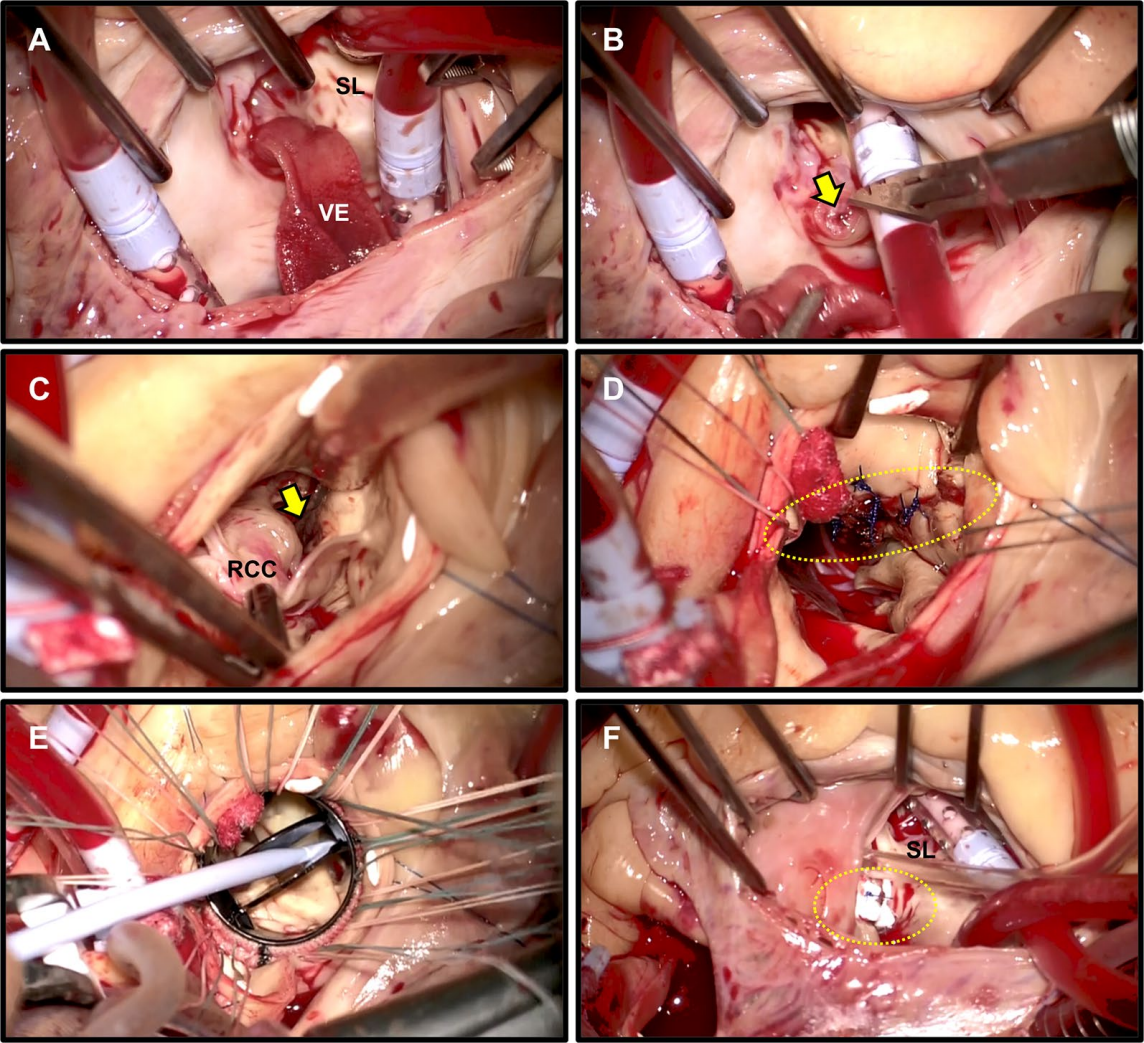
Intraoperative images. **A** Large vegetation attached to the atrioventricular septum above the tricuspid septal leaflet. **B** Gerbode defect was revealed following the removal of the vegetation (yellow arrow in **B**). **C** Right coronary cusp detachment from the aortic annulus. The abnormal cavity was visible through a tear in the LVOT (yellow arrow in **C**). **D** LVOT repair by direct closure following the removal of the aortic valve (yellow dotted circle in **D**). **E** Aortic valve replacement using a mechanical aortic valve. **F** Defect closure by placing a pledgeted mattress suture from the RA side (yellow dotted circle in **F**). SL: septal leaflet; VE: vegetation; RCC: right coronary cusp

The patient underwent urgent surgery the day after admission to our hospital. Standard full sternotomy was performed, and cardiopulmonary bypass was established via ascending aorta perfusion and bicaval drainage. Through a right atriotomy, a large vegetation attached to the atrioventricular septum above the tricuspid septal leaflet was identified (Fig. 2A). It was also confirmed that the tricuspid valve was not infected. A perforation of the atrioventricular septum was revealed following the removal of the vegetation (Fig. 2B). Following aortotomy, the right coronary cusp of the aortic valve was completely detached from the annulus, as shown in the preoperative TEE (Fig. 2C). Additionally, it was found that the left ventricular outflow tract (LVOT) had been torn. The abnormal small cavity was visible through a tear in the LVOT (Fig. 2C). After removing the aortic valves, a cavity filled with infected granulation tissues and penetrating to the RA was found. We subsequently debrided the infected tissues completely and the LVOT was repaired by direct closure using 4–0 monofilament interrupted sutures (Fig. 2D). Next, aortic valve replacement (AVR) was performed using a 20-mm ATS AP360 mechanical aortic valve (Medtronic Medical, Santa Rosa, CA) (Fig. 2E). Subsequently, the Gerbode defect was closed securely by placing a 4-0 pledgeted monofilament mattress suture from the RA side (Fig. 2F). Postoperative TTE showed favorable prosthetic aortic valve function with no perivalvular leakage or residual shunt blood flow. Intravenous antibiotics were continued postoperatively for 6 weeks. The patient was uneventfully discharged without any complications, including an atrioventricular block. The patient has remained in good health for 4 years after surgery, with recent echocardiography indicating no recurrence of IE or LV–RA shunt.

## Discussion

Gerbode and colleagues first described a case series of successful surgical closure for an LV–RA defect [2]. This defect is a rare intracardiac abnormality caused by a deficiency in the membranous ventricular septum that separates the LV from the RA. Gerbode defect is usually congenital, but can also be acquired as a complication of IE, myocardial infarction, blunt chest trauma, or previous cardiac surgery [3]. In cases associated with IE, the infection often extends into the aortic subannular region, involving the high membranous septum. This leads to the rupture of the portion of the septum and results in an LV to RA shunt with an intact tricuspid valve [4].

Compared with the more stable congenital Gerbode defects, acute fistulation between the LV and the RA associated with IE is a life-threatening complication that often requires urgent definitive intervention [5]. However, the identification of actual communication is often extremely difficult. Small acquired Gerbode defects are usually asymptomatic and can be easily missed in TTE. Even in our case, the defect could not be detected by TTE. TEE has been demonstrated to be superior to TTE in the detection of vegetations associated with endocarditis and subaortic complications such as fistula formation [6]. In our case, by detecting the presence of the abnormal cavity immediately below the right coronary cusp and in contact with RA using TEE, the possibility of LV–RA communication was suspected. Additionally, the location of the vegetation attachment, not to the leaflet of the tricuspid valve but to the atrioventricular septum, the site of a potential Gerbode defect, was another finding that led to suspicion of a defect. Through careful TEE examination, the Gerbode defect was finally detected. Especially if the defect is small and not visible easily on the TTE, it is important to suspect the presence of a defect from these secondary findings and to perform a careful and meticulous TEE for accurate early diagnosis and successful repair.

In most cases, a direct suture from the RA side is sufficient to close the defect [3, 4]. However, in cases of large defects with extensive tissue destruction, defect patch closure and the reconstruction of the tricuspid valve and/or LVOT besides AVR are mandatory [3, 7]. In our case, a part of the LVOT wall was destroyed, but the infection did not extend extensively, and could be simply repaired by direct suture without using a patch. If the diagnosis had been delayed, the LVOT could have been more extensively destroyed or an LVOT pseudoaneurysm could have formed and required patch repair, which could be much more challenging.

Our case was unusual in that *S. agalactiae*, a type of GBS was the causative organism of IE. GBS is a beta-hemolytic Gram-positive bacteria that is known to be an important causative organism of sepsis in pregnancy and neonates but has been an uncommon cause of IE. However, GBS IE has recently been increasingly reported [1]. The clinical course of patients with GBS IE tends to progress rapidly and is more likely to be complicated by stroke, systemic embolization, and acute congestive heart failure because it forms large, friable vegetations and has a markedly destructive effect on valvular tissue. Early surgical intervention is necessary to prevent the rapid progression of the disease and reduce mortality. In studies that report data collected after 2000, mortality has ranged from 20 to 33.3% [8]. Although the mortality rate has been lower than in the past due to increased recognition, improved diagnosis, and early surgical intervention, it remains high. In addition, diabetes in particular is a risk factor for the development of invasive GBS disease, consistent with the patient in this case. The presence of GBS bacteremia, especially among people who have a risk factor such as diabetes, should prompt clinicians to have a high suspicion of IE.

## Conclusions

We reported a case of unusual active IE and Gerbode defect caused by GBS that was successfully treated by direct defect closure, LVOT repair, and concomitant AVR. Careful preoperative echocardiographic work-up is imperative for accurate early diagnosis and successful repair.

## Data Availability

The datasets used in this case report are available from the corresponding author on reasonable request.

## Abbreviations

LV: Left ventricle
RA: Right atrium
IE: Infective endocarditis
GBS: Group B *Streptococcus*
TTE: Transthoracic echocardiography
AR: Aortic regurgitation (AR)
TEE: Transesophageal echocardiogram
LVOT: Left ventricular outflow tract
